# Habit History in Oral Submucous Fibrosis: Have We Over Emphasized?

**DOI:** 10.31557/APJCP.2019.20.2.451

**Published:** 2019

**Authors:** Sheshaprasad R, Anuradha Pai, Anisha Yaji

**Affiliations:** 1 *Department of Oral Medicine and Radiolog , The Oxford dental College,*; 2 *Department of Oral Medicine and Radiology, The Oxford Dental College and Hospital Bommanahalli Hosur Road,*; 3 *Consultant Oral Medicine and Radiologist Sri krishna sevashrama Hospital Jayanagar 5th Block, Bengaluru, India.*

**Keywords:** Arecanut, correlation, Habit, OSMF

## Abstract

**Aim:**

Correlation of habit duration and frequency with clinical grading and histopathologic grouping of oral submucous fibrosis (OSMF).

**Materials and methods:**

48 patients with OSMF were included in the study. Detailed history of each patient, clinical profile and habit history were recorded. Biopsy was performed for histopathological correlation. All the findings were correlated with Kerr et al and Khanna and Andrade classification.

**Statistical Analysis:**

Kruskal-wallis test was performed to assess the correlation between the study findings.

**Results:**

Out of 48 OSMF cases majority were males. Maximum cases were in clinically and histopathologically respectively. Mouth opening was directly proportional with histopathological grouping as per Khanna et al. No correlation between frequency and habit duration with respect to different stages or severity of the OSMF was noted.

**Conclusion:**

Disease staging of OSMF clinically and histopathologically is not directly impacted by habit duration and frequency. Rather than habit centered history and management accordingly, more focus should be given to genetics and susceptibility of patient for OSMF development and progression.

## Introduction

OSMF is predominant among the people of South Asia and is closely associated with the habit of areca nut chewing. Evidence shows that areca nut greatly contributes towards exaggerating the occurrence of OSMF (Goel et al., 2010). The prevalence of OSMF in 2010 was reported to be 6.42% with an estimate of 5 million Indians suffering from this condition (Nigam et al., 2004). Oral fibrosis is caused due to increased synthesis of collagen, and in turn induces the production of free radicals and reactive oxygen species. This leads to high rate of oxidation/peroxidation of polyunsaturated fatty acids which affect essentials of cell membrane, which may induce tumorigenesis (Ajagaokar et al., 2016). Since a lot of emphasis is laid on the arecanut habit in contribution of OSMF, this study was under taken to assess the impact of habit frequency and duration with clinical staging and histopathological grading.

## Materials and Methods

*Methodology*

The present study was carried out in the department of Oral Medicine and Radiology, the Oxford Dental College and Hospital, Bangalore. The ethical board clearance was obtained. 48 subjects diagnosed with OSMF who consented to participate were included in the study. Clinical details including name, age, gender and habits history along with duration of habit in years, frequency of habit per day, style of chewing i.e. spitting, swallowing and duration taken to chew was recorded on pre decided proforma. Mouth opening was measured using Vernier caliper. Clinical Grading of the lesion was performed using Kerr et al., (2011) classification (Table-1). After that a biopsy was taken from subjects for histopathologic grouping by Ranganathan and Mishra (2006) (Table-2).

*Statistical analysis*

The data obtained were tabulated and analysed using SPSS software version SPSS v.22 software IBM., Corp.

Comparison of mean duration and frequency of habit between different of OSMF clinically and histopathologically were done using Kruskal Wallis Test.

**Figure 1:**
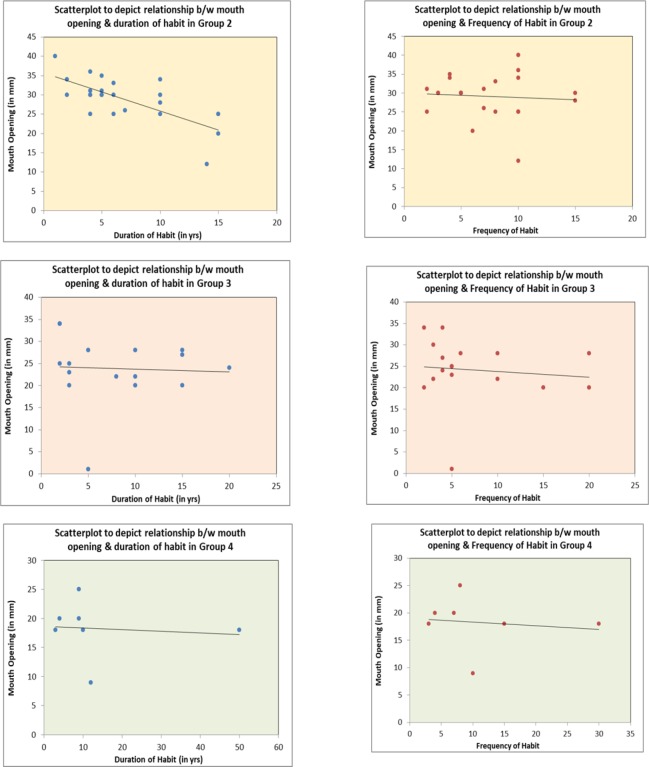
Scatterplot Depicting Relation between Frequency and Duration of Habit with Different Clinical Groups of OSMF

**Table 1:** Kerr e al., (2011) Classification

Grade 1– Mild	Any features of the disease triad for OSMF (burning, depapillation, blanching or leathery mucosa) may be reported – and inter-incisal opening >35 mm
Grade 2 – Moderate:	Above features of OSMF + inter-incisal limitation of opening 20–35 mm
Grade 3 – Severe:	Above features of OSMF + inter-incisal opening <20 mm
Grade 4A –	OSMF + other potentially malignant disorder on clinical examination
Grade 4B –	OSMF with any grade of oral epithelial dysplasia on biopsy
Grade 5 –	OSMF + oral squamous cell carcinoma (SCC)

**Table 2:** Khanna and Andrade., (1995) Classification

Group	Clinical	Histology
Group I: Very early cases:	Common symptom is burning sensation in the mouth, acute ulceration and recurrent stomatitis and not associated with mouth opening limitation.	Fine fibrillar collagen network interspersed with marked edema, blood vessels dilated and congested, large aggregate of plump young fibroblasts present with abundant cytoplasm, inflammatory cells mainly consist of polymorphonuclear leukocytes with few eosinophils. The epithelium is normal.
Group II: Early cases	Buccal mucosa appears mottled and marble like, widespread sheets of fibrosis palpable, interincisal distance of 26 to 35 mm.	Juxta-epithelial hyalinizalion present, collagen present as thickened but separate bundles, blood vessels dilated and congested, young fibroblasts seen in moderate number, inflammatory cells mainly consist of polymorphonuclear leukocytes with few eosinophils and occasional plasma cells, flattening or shortening of epithelial rete-pegs evident with varying degree of keratinization.
Group III: Moderately advanced cases	Trismus, interincisal distance of 15 to 25 mm, buccal mucosa appears pale firmly attached to underlying tissues, atrophy of vermilion border, vertical fibrous bands palpable at the soft palate, pterygomandibular raphe and anterior faucial pillars.	Juxta-epithelial hyalinization present, thickened collagen bundles, residual edema, constricted blood vessels, mature fibroblasts with scanty cytoplasm and spindle-shaped nuclei, inflammatory exudates which consists of lymphocytes and plasma cells, epithelium markedly atrophic with loss of rete pegs, muscle fibers seen with thickened and dense collagen fibers.
Group IVA: Advanced cases	severe trismus, interincisal distance of less than 15 mm, thickened faucial pillars, shrunken uvula, restricted tongue movement, presence of circular band around entire lip and mouth.	Collagen hyalinized smooth sheet, extensive fibrosis, obliterated the mucosal blood vessels, eliminated melanocytes, absent fibroblasts within the hyalinised zones, total loss of epithelial rete pegs, presence of mild to moderate atypia and extensive degeneration of muscle fibers.
Group IVB: Advanced cases	presence of hyperkeratotic leukoplakia and/or squamous cell carcinoma.	

**Table 3:** Distribution of Sociodemographic Characteristics among Study Patients

Variable		n	%
Sex	Males	39	81.20%
	Females	9	18.80%
Age Group	20-30 yrs	31	64.60%
	31-40 yrs	11	22.90%
	41-50 yrs	2	4.20%
	51-60 yrs	1	2.10%
	>60 yrs	3	6.30%
Age	Mean and SD	32.4	12
	Range	20 - 80	

**Table 4:** Comparison of Mean Duration (in yrs) and Frequency (Pkts/ day)of Gutkha Intake between Different Grades of OSMF as per Kerr et al Grading System Using Kruskal Wallis Test

Variables /Grades	N	Mean	SD	Min	Max	H	P-Value
Duration							
Grade 1	1	1	..	1	1	6.205	0.1
Grade 2	40	7.4	4.8	1	20		
Grade 3	6	5.7	4.1	3	14		
Grade 5	1	50	..	50	50		
Frequency							
Grade 1	1	10	..	10	10	2.886	0.41
Grade 2	40	7.7	5.9	2	30		
Grade 3	6	6.2	3.1	3	10		
Grade 5	1	15	..	15	15		

**Table 5:** Comparison of Mean Mouth Opening Measurement (in mm) among the Study Patients between Different HP Grouping as per Khanna and Andrade et al Grouping System Using Kruskal Wallis Test

Variables / Groups	N	Mean	SD	Min	Max	H	P-Value
Mouth Opening							
Group 1	1	32	..	32	32	18.012	<0.001*
Group 2	23	29.1	5.8	12	40		
Group 3	17	24.2	7.4	1	34		
Group 4	7	18.3	4.8	9	25		

**Table 6:** Different Classifications Used in Studies and Their Findings

Study	Participnts	Classification used	Positive Habit and disease severity corelation	Positive clinical and histopathological Correlation
Goel et al	100	Kiran kumar et al	yes	No
Kumar et al	75	Kiran kumar et al		No
Debanath et al	100	Kiran kumar et al	No	No
Pandya S et all	239	Pindborg and Sirasat	Yes	No
Ali et al	197	Ranganathan et al	Yes	-
Shivakumar et al	50	Kerr etal and Khanna etal	Yes	yes

## Results

This study included a total of 48 subjects with OSMF out of which 81.2% were males and 18.8% females. Majority of the subjects were in the age group of 20 -30 years (64.6%) and 6.3% of them above 60 years. (Table 3)

A comparison between mean habit duration and frequency with grading of mouth opening using Kerr et al classification was done. Maximum subjects (n=40) were in grade 2 with a mouth opening of 20-35 mm. with a mean of 7.4 years of habit duration and a frequency ranging from 1 time to 20 times per day.(Table 4) Grade 1 and 5 had only one subject with 50 years and 1 year of duration respectively. The correlation between the habit duration and frequency with disease severity was not significant. When the clinical symptom was correlated with histopathologic grading, Group 2 had maximum subjects (N=17) and group 1 minimum (N=1) according to Khanna and Andrade et al classification. (Table 5) Group 4 cases had least mouth opening of 18.2 mm as compared to group 2 which had a mouth opening of 29.1mm. As the mouth opening decreased the histopathological grouping increased. The correlation between mouth opening and histopathologic grading was statistically significant (p= <0.001). 

## Discussion

OSMF is a fibrotic disorder characterized by a presence of chronic low grade irritant i.e arecanut (Gandhi et al., 2017). Apart from this arecoline content in arecanut is also known to cause impaired endothelial function. Its effect on p53 inhibition, DNA repair suppression and DNA damage triggering in response to human endothelial cells have also been studied (Tseng et al., 2012). Many studies have emphasised on cessation of the areca nut chewing habit as the mainstay for the therapy (Prabhu et al., 2014; Bhatnagar et al., 2018). Angadi et al., (2011) suggested at a different mechanism of action of arecoline by increased differentiation of myofibroblasts in OSMF from various sources rather than a dose related pathogenesis. Also there was disagreement between studies regarding the relation between the development of OSMF and habit frequency and duration. Hence this study was under taken with the primary aim of assessing the impact of duration and frequency of habit with the clinical and histological expression of disease. The secondary aim of this article was addition of the demographic data with diverse use of areca nut to global OSMF data.

This study had a male predominance with majority (64.6%) of them in the age group of 20 -30 years. Multiple classification systems have been used for clinical and histological grading of the OSMF (Ranganathan et al., 2006). In our study Kerr et al., (2011) classification was considered as this is one of the few classifications which include dysplasia. However no cases with dysplasia were seen in this study. Histopathologic classification of Khanna and Andrade was considered as Kerr et al classification did not consider histopathological changes other than dysplasia. 

This study we observed that the clinical grades (mouth opening) had no statistically significant direct correlation with the habit duration or frequency. Ali et al., (2013) study on 197 subjects, Pandya et al., (2009) study which included 239 patients, Goel et al., (2011) study which included 100 patients and Shivkumar et al., (2010) study concluded that duration and frequency of areca nut use and had a direct effect on the incidence and severity of OSMF unlike our study (Table 6). Seedat et al., (2013) and Debanth et al., (2013) study similar to our study had noted that there was no positive correlation between habit duration and frequency and expression of disease. These studies concluded that chewing areca nut will lead to pathological changes in the mucosa, but, neither the frequency nor the duration of the habit, are accurate predictors of the extent of these changes or when they are likely to occur, which is clearly seen in our study. We also noted OSMF development (n=7) 3-4 years after cessation of habit by patients. As we only included patients who were active habit users we did not include them in the study.

When the clinical grading was compared with histopathologic grouping there was significant correlation between the two. This was not in accordance with other studies with the exception of one study i.e Shivkumar et al., (2010) study (Goel et al.,2010; Debnath et al., 2013; Kumar et al., 2007). Goel et al., (2010) attributed this variation to the possibility of difference in the severity and extent of ﬁbrosis in different regions of the oral mucosa and involved muscles. 

However it’s difficult to debate about the deeper tissue changes as our study used a histopathological grouping which limited its field of interest only to the subepithelial layers. 

The difference in correlation between habit duration, frequency and disease progression may be largely attributed to genotoxic effects like polymorphisms of genes associated with collagen homeostasis brought about by exposure to habit (Shivkumar et al., 2010). This also explains why some patients though they have a shorter habit duration have severe OSMF as compared to their longer duration counter parts. Hence, when patients with arecanut habit have symptoms of OSMF, a long term follow up is warranted irrespective of its stage or grade. Habit cessation alone may not ensure prevention of disease incidence or progression.

*Limitations of our study*

1. We solely depended on patients reporting of habits for habit history. 

2. We had not considered patients who had developed OSMF even after discontinuity of any tissue abusing products like arecanut, tobacco or alcohol for more than a year previously. Such patients also should be included as there have been incidences of lesion development as late as 10 years post discontinuation of habit. 

3. There were severe differences in the number of patients in each group. A larger sample size would have probably eliminated this issue.

4. There is no fixed protocol for biopsy site selection in OSMF. This could have contributed to variation in histologic expression of disease. 

In conclusion, this study shows that frequency and duration of habit showed no correlation with disease severity. Also in future more samples should be recruited from multiple centers with correlation of deeper tissue structure including muscular changes if any. Rather than habit centered history and management, more focus should be given to genetics and susceptibility of patient for OSMF development and progression. Also, when patients with initial stages of OSMF are encountered they must be emphasized and educated with the importance of long term follow up after habit cessation.
